# Antioxidant and Hypolipidemic Activity of Açai Fruit Makes It a Valuable Functional Food

**DOI:** 10.3390/antiox10010040

**Published:** 2020-12-31

**Authors:** Anna Virginia Adriana Pirozzi, Paola Imbimbo, Antonella D’Agostino, Virginia Tirino, Rosario Finamore, Daria Maria Monti, Renata Piccoli, Chiara Schiraldi

**Affiliations:** 1 Department of Experimental Medicine, Section of Biotechnology, Medical Histology and Molecular Biology, University of Campania “Luigi Vanvitelli”, via De Crecchio 7, 80138 Naples, Italy; adripirozzi@gmail.com (A.V.A.P.); antonella.dagostino@unicampania.it (A.D.); virginia.tirino@unicampania.it (V.T.); rosario.finamore@unicampania.it (R.F.); 2 Department of Chemical Sciences, University of Naples Federico II, via Cinthia 21, 80126 Naples, Italy; paola.imbimbo@unina.it (P.I.); mdmonti@unina.it (D.M.M.); renata.piccoli@unina.it (R.P.)

**Keywords:** HepG2 cells, oxidative stress, steatosis, NAFLD, açai, MDA, NF-kB, PPAR α/γ

## Abstract

Several plant extracts are acquiring increasing value because of their antioxidant activity and hypolipidemic properties. Among them, great interest has been recently paid to açai fruit as a functional food. The aim of this study was to test the ability of açai extract in reducing oxidative stress and modulating lipid metabolism in vitro using different cell models and different types of stress. In fact, lipid peroxidation as evaluated in a HepG2 model was reduced five-fold when using 0.25 µg/mL of extract, and it was further reduced (20-fold) with the concentration increase up to 2.5 µg/mL. With the nonalcoholic fatty liver disease (NAFLD)in vitro model, all concentrations tested showed at least a two-fold reduced fat deposit. In addition, primary adipocytes challenged with TNF-α under hypoxic conditions to mimic the persistent subcutaneous fat, treated with açai extract showed an approximately 40% reduction of fat deposit. Overall, our results show that açai is able to counteract oxidative states in all the cell models analysed and to prevent the accumulation of lipid droplets. No toxic effects and high stability overtime were highlighted at the concentrations tested. Therefore, açai can be considered a suitable support in the prevention of different alterations of lipid and oxidative metabolism responsible for fat deposition and metabolic pathological conditions.

## 1. Introduction

The fruit of açai tree (*Euterpe oleracea Martius*) is a Brazilian berry found in the Amazon flood plain and usually consumed in South America [1]. In recent years, this exotic fruit, traditionally used as a medicinal plant and as a staple food, has gained international attention as a functional food due to its nutritional benefits and therapeutic promises [2]. In fact, açai contains high amount of α-tocopherol, dietary fibres, lipids, polyphenols (including anthocyanins), and mineral ions [2,3]. Most of the beneficial effects of açai are attributed to secondary metabolites, such as anthocyanins and proanthocyanidins (specifically cyanidin 3-O-glucoside and cyanidin 3-O-rutinoside) [3,4]. We recently reported the antioxidant activity of the açai extract on immortalized fibroblasts (BALB/c 3T3) and found that the protective activity was due to two anthocyanins, cyanidin and malvidin derivatives [3,5,6]. Indeed, the extract had the same antioxidant activity of the isolated molecules [3]. Besides the antioxidant activity, anti-inflammatory [7], anti-cancer, immunomodulatory, and antinociceptive [8] activity have been reported. As for the anti-cancer activity of açai extract, the MTT assay on lung cancer cells (A549) showed a maximum decrease (about 70%) in cell viability after 48 h treatment with açai (200 µg/mL) and a strong increase in the number of apoptotic cells when compared to untreated cells [9]. Oliveira de Souza and collaborators (2010) [10] also reported the hypolipidemic effects of açai, and more recently açai was tested for its activity on the modulation of hepatic markers of steatosis and on the increment of serum activity of antioxidant enzymes in steatotic in vivo models [11]. The extract was also found to reduce the adiposity in obesity mice [12]. Although several reports were published on the spectrum of antioxidant and hypolipidemic benefits of açai, only few studies are available so far on the identification of the mechanism responsible for the foreseen beneficial effects on human health [13]. Obesity is considered a chronic disease that affects people (about 50% in the western world) independently of age, ethnicity, or class [14]. As two pathological fat accumulation processes, i.e., steatosis and chronic abdominal fat deposition, are involved in the development of obesity, inducing oxidative impairment and inflammation state, we investigated the effects of açai extract in not alcoholic hepatic steatosis (NAFLD) and persistent subcutaneous fat accumulation in vitro. NAFLD is considered one of the most predominant liver diseases worldwide, but no specific treatments have been found [15]. However, it has been reported that phenolic compounds, especially those belonging to the anthocyanin class, are able to help in the prevention and/or treatment of the disease. As a genotoxic assessment revealed the absence of significant DNA and chromosome damage in human HepG2 cells when treated with açai extract [16], we used HepG2 cells to mimic steatosis [17], and primary adipocyte cells, treated with TNF-α, as a model of persistent subcutaneous fat accumulation induced by an inflammatory state in vitro [18]. In the present study, we analyzed the effect of açai extract on the reduction of inflammatory states, highlighted its ability to modulate oxidative stress in combination with fat deposit accumulation, and revealed its potent effect to reduce key biomarkers related to fat accumulation.

## 2. Materials and Methods

### 2.1. Reagents

All the reagents, unless differently specified, were obtained from Sigma Aldrich (Milan, Italy). Antibodies against NF-kB (C-20, sc-37), PPARα (H-98, sc-9000), β-actin (I-19, sc-1616), and monoclonal antibodies against PPARγ (E-8, sc-7273) were from Santa Cruz Biotechnology (Santa Cruz, CA, USA). Antibodies anti-CD34 PE were from Miltenyi-Biotech; antibodies anti-CD90 FITC, anti-CD105 APC, anti-CD29 PerCP Cy5-5, anti-CD31 FITC, anti-CD44 PerCP Cy5-5, anti-CD45 APC-Cy7 and anti-CD14 BD HORIZON V500 were from BD Pharmingen. All cell culture materials were purchased from Gibco (Invitrogen, Milan, Italy).

### 2.2. Cell Selection and Culture

Immortalized human keratinocytes (HaCaT) cells were from Innoprot (Derio—Bizkaia, Spain). HepG2 cell line was provided by ATCC Cell Biology Collection cells. Cells were cultured in Dulbecco’s modified Eagle’s medium (DMEM) supplemented with 10% FBS, 100 U/mL penicillin, 100 μg/mL streptomycin and 100 μg/mL antifungal. HaCat medium was also added with glutamine (2 mM). All cell culture materials were purchased from Gibco (Invitrogen, Milan, Italy). The cells were grown on tissue culture plates (BD Bioscience-Falcon, San Jose, CA, USA), in a humidified atmosphere (95% air and 5% CO^2^, *v*/*v*) at 37 °C. Human adipose-derived stem cells (hASCs) isolated from adipose tissue obtained by lipectomy or liposuction in the Plastic and Reconstructive Surgery Clinic of University of Campania “L. Vanvitelli” were grown as previously reported by Stellavato et al. 2017 [18]. For cell culture experiments, açai isopropanol extract was suspended in PBS at 0.25 mg/mL.

### 2.3. Açai Extracts Preparation

Ethanolic and isopropanolic extracts from açai fruit were obtained as reported by Petruk et al. 2017 [3], with some modifications. Briefly, 2 g of dried powder were extracted with 25 mL of pure ethanol or pure isopropanol using an ultrasonic bath (Branson 5200 Ultrasonic Corp.) for 60 min on ice. The mixture was centrifuged at 3000× *g* for 10 min at 4 °C. The supernatants were removed and collected. Solvents were removed, dried under N_2_ stream and weighted. Then, 0.25 mg were recovered after drying, suspended in H_2_O or PBS (0.25 mg/mL) and stored under different experimental conditions: 4 °C, –20 °C, 37 °C, or at room temperature (either exposed to light or stored in the dark).

### 2.4. HPLC Analyses

500 mg of dry extract was solubilized in 1 mL of methanol (99,8% purity, VWR, Milan) for 1 h and then centrifuged for 10′ at rpm 1200 on a Centrifuge 5415R (Eppendorf, Hamburg, Germany). The supernatant was analysed by HPLC using a C18 reverse phase column (EC250/4.6 Nucleodur 100-5 C18ec Macherey-Nagel, (Delchimica Scientific Glassware s.r.l., Naples, Italy) and an ultra-pressure liquid chromatography system (UPLC Ultimate 3000^+^, Thermo Fisher Scientific, Italy), equipped with an UV detector (Wavelenght: 280 nm). The buffers used were obtained dissolving in ultrapure water (HPLC-grade). Buffer A was 0.1% *v*/*v* trifluoroacetic acid (TFA) and buffer B was 0.1% TFA in acetonitrile. 20 µL of the sample were injected and eluted in gradient conditions at a flow rate of 0.2 mL·min^−1^ (0–5 min at 5% B, 5–45 min from 5% to 95% B, 45–50 min at 95% B, 50–55 min from 95% B to 5% B; 45–50 min at 5% B) at 40 °C.

### 2.5. 2′,7′-dihydrodichlorofluorescein Diacetate Assay (DCFDA)

To analyze the stability of açai extract as antioxidant, intracellular ROS levels was measured by DCFDA assay on HaCaT cells after 0, 3, 7, 14, 21, and 70 days. Briefly, cells were seeded at 2.5 × 10^4^ cells/well. Then, 24 h after seeding, 1.25 µg/mL of extracts stored in different conditions –20 °C, 4 °C or r.t., in dark or under light exposure, at 37 °C), were added to the culture medium. After 2 h incubation, cells were treated with 25 µM 2′,7′-dihydrodichlorofluorescein diacetate (H_2_-DCFDA, Sigma-Aldrich) for 45 min at 37 °C in complete medium without phenol red. At the end of incubation, cells were incubated with 300 µM NaAsO_2_. Then, cells were washed with warm PBS plus (phosphate buffer saline supplemented with 1 mM CaCl_2_, 0.5 mM MgCl_2_, and 30 mM glucose). The fluorescence intensity of the DCF probe was measured by using a Synergy™ HTX Multi-Mode Microplate Reader (excitation = 485 nm, emission = 535 nm, scanning speed = 300 nm/min and 5 slit widths for excitation and emission, BioTek Instruments, Inc., Winooski, VT, USA). ROS production was expressed as the DCF fluorescence intensity of the samples under testing with respect to untreated cells. The mean ± standard deviation (SD) was calculated from at least 3 experiments each with triplicate measurements.

### 2.6. Cell Viability Assay

Cytotoxicity was assessed in HepG2 and adipocyte cells (1.0 × 10^5^ cells/well in a standard 24-well culture plate) using Presto Blue^®^ from Thermo Fischer Scientific. The PB assay is a commercially available, ready-to-use, water-soluble preparation. Cells were incubated for 24 h with a 6 mM mixture of fatty acids (FAs) ratio 1:1 *v*/*v* (L9655-Sigma Aldrich) and then treated with the açai extract (0.25 mg/mL) at: 0.25, 0.625, 1.25, 2.5 µg/mL final concentration. After 24 h, the cells were incubated with Presto Blue reagent. Cell viability was measured spectroscopically (absorbance at 570 nm with a reference wavelength set at 600 nm) using a Bio-Rad multiwell plate reader (Bio-Rad Laboratories, Milan, Italy) and expressed as the percentage relative to the viability of cells untreated with the compound.

### 2.7. Lipid Peroxidation Assay

HepG2 cells (1 ×  10^5^ cells/cm^2^) were prepared with 6 mM of a mixture of fatty acids (FAs) based on linoleic acid (18:2, ω-6 fatty acid) and oleic (18:1, ω-9 fatty acid) in ratio 1:1 *v*/*v* (L9655-Sigma Aldrich) for 24 h and thus pre-treated for 30 min with 50 µM H_2_O_2_ and then incubated with the açai extract at different concentrations (0.25, 0.625, 1.25, 2.5 µg/mL) for 24 h. HepG2 not exposed to FA were treated for a control with 50 µM H_2_O_2_ for 30 min, the protein concentration of cell lysates was determined using the Bio-Rad protein assay reagent (Bio-Rad Laboratories, Milan, Italy). Lipid peroxidation was evaluated by quantifying thiobarbituric acid reactive substances (TBARS) as reported by Stellavato et al. (2018). The data represent the reduction percentage with respect to cells treated with H_2_O_2_. The mean ± standard deviation (SD) was calculated from 3 experiments, each with triplicate measurements.

### 2.8. In Vitro Steatosis and Determination of Intracellular Lipid Content

In vitro steatosis [17,19,20] was induced as described in Section 2.7, by incubating HepG2 cells (1.0 × 10^5^ cells/well seeded in a standard 24-well culture plate) with FA. After a PBS washing, cells were exposed for an additional 24 h to different concentrations (0.25, 0.625, 1.25, 2.5 µg/mL) of açai extract for 24 h. Lipid amount was determined using Oil Red O solution (0.5% *v*/*v*). Cells pictures were captured by an optical microscope and stained lipid droplets were then extracted with isopropanol (60% *v*/*v*) and quantified by measuring the absorbance at 510 nm [19].

### 2.9. Fat Deposition in Hypoxic Condition Using Adipocytes In Vitro Model

Adipocytic cells (3.0 × 10^5^) were cultivated in hypoxic conditions, treated with TNFα (10 ng/mL) alone (damaged/stress condition), or in combination with açai extract at different concentrations (0.25, 0.625, 1.25, 2.5 µg/mL) for 24 h to evaluate potential its beneficial effect in rescuing the stressful condition. We used cobalt chloride-induced chemical hypoxia and a hermetically sealed box containing activated carbon to sequester the residual oxygen, as previously reported [15]. The control (physiological condition) was represented by cells cultured in normal condition of oxygen.

### 2.10. Western Blotting Analyses

At the end of each treatment, proteins were extracted using RIPA and NE/PER lysis buffers. Protein content was determined using the Bio-Rad protein assay reagent (Bio-Rad Laboratories, Milan, Italy). From each sample, 30 µg of proteins were loaded on a SDS-PAGE and transferred to a nitrocellulose membrane. Filters were incubated with antibodies against NF-kB (1:200 *v*/*v*), PPARα (1:250 *v*/*v*), PPARγ (1:250 *v*/*v*) or β-actin (1:500 *v*/*v*) at room temperature for 2 h. Membranes were washed three times for 10 min with TBS Tween 20 and incubated with a 1:10,000 dilution (*v*/*v*) of horseradish peroxidase-conjugated anti-mouse, anti-rabbit or anti-goat antibodies, respectively, for 1 h as previously reported [17]. Blots were developed using an ECL system (Amersham Biosciences) according to the manufacturer’s protocols.

### 2.11. Statistical Analysis

All the experimental results were expressed as the mean ± standard deviation (SD) of at least three independent determinations for each experiment. Deviation statistical significance were assessed by Student’s *t* tests. *p* values * < 0.05 and ** < 0.01 were considered significant. When statistical analysis is indicated by letters, the significance is reported in the legend.

## 3. Results

### 3.1. HPLC Analyses

**Figure 1:**
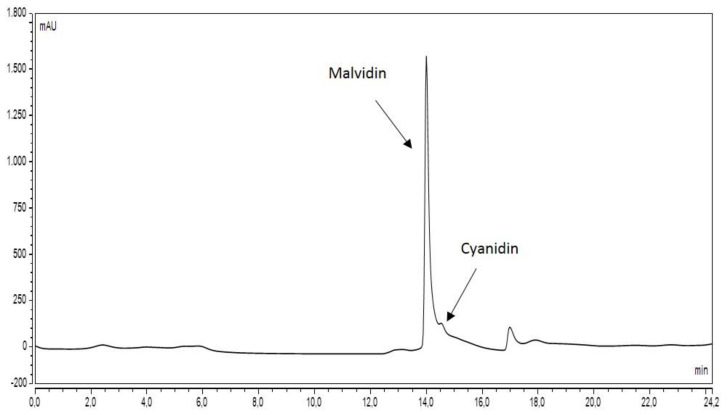
Chromatographic profile of açai extract by reverse-phase HPLC. We identify two main peaks that have the same retention times of chromatagram reported by Petruck et al. The two species can be malvidin and cyanidin, the active principles of açai extract.

The elution of açai extract obtained as described in the Methods section showed a profile similar to the one reported by Petruk et al. [3]. The elution time of the peak known to be representative for the bioactive molecules (malvidin and cyanidin, [3]) is consistent in our chromatographic profile (Figure 1) respect to the published data. Only minor changes in the profile were evidenced for the main peak area, that were possibly due to the different dimension of the column section and the consequently a higher flow rate, and also a higher methanol content in the loaded sample.

### 3.2. Antioxidant Activity of Açai Extract on HaCaT Cells

We first evaluated the antioxidant activity of açai extract and its stability in different buffers and in different experimental conditions. A cell-based model was used to test the antioxidant activity of the samples stored in different experimental conditions (–20 °C, +4 °C, r.t in dark or under light exposure, 37 °C), up to 10 weeks. Immortalized human keratinocytes (HaCaT cells) were pretreated with 1.25 µg/mL of each extract for 2 h, and then oxidative stress was induced by adding 300 µM sodium arsenite (SA) for 1 h. Results are reported in Table 1, as percentage of ROS levels with respect to untreated cells (cells in which the physiological release of ROS is measured). In each set of experiments, cells were also incubated only in the presence of SA (last column of Table 1). In a parallel experiment, açai was solubilized in methanol, and used as internal control, as the antioxidant activity with methanol extract has been already reported (sample in methanol was 118 ± 7%) [3]. As shown in Table 1, after solubilization, most of the samples were found to inhibit ROS formation, as the % obtained were found to be generally lower than the % measured in the SA treated cells. From a general point of view, it seems that the extract is more stable when stored at room temperature, with respect to –20 or 37 °C.

#### 3.2.1. Effects of Açai Extract on HepG2 Cell Viability

**Figure 2:**
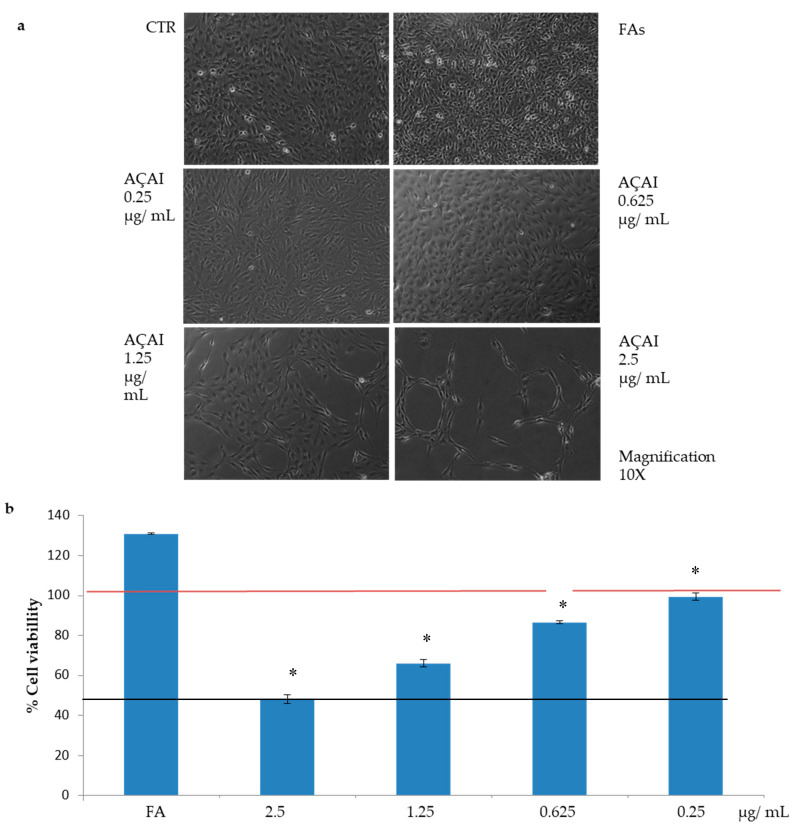
Viability of steatotic HepG2 cells in the presence of açai extract. After 24 h of FAs incubation, cells were treated with increasing concentrations of açai extract (from 0.25 to 2.5 µg/mL) for other 24 h. (**a**) Images of cells obtained by optical microscopy, 10× magnification; (**b**) Viability, assessed by Presto Blue reagent, is expressed as percentage with respect to untreated cells (indicated by the red line). The black line indicates the 50% of cell viability. * *p* < 0.05 for all the acaj added samples vs FA. Treatment with 0.25 µg/mL acaj extract proved the same cell viability than untreated control. Only 2.5 µg/mL treatment reduced viability of 50% respect to control, showing a certain cytotoxicity.

Steatotic HepG2 cells showed a dose dependent reduction in cell viability (Figure 2a,b). The highest concentration tested (2.5 µg/mL) was found to induce a 50% decrease in cell viability, whereas no significant toxicity was observed at lower concentrations. This result is not surprising as a similar behavior was observed with other well-known antioxidant natural extracted molecules [15].

#### 3.2.2. Effects of Açai Extract on Lipid Peroxidation in HepG2 Cells

**Figure 3:**
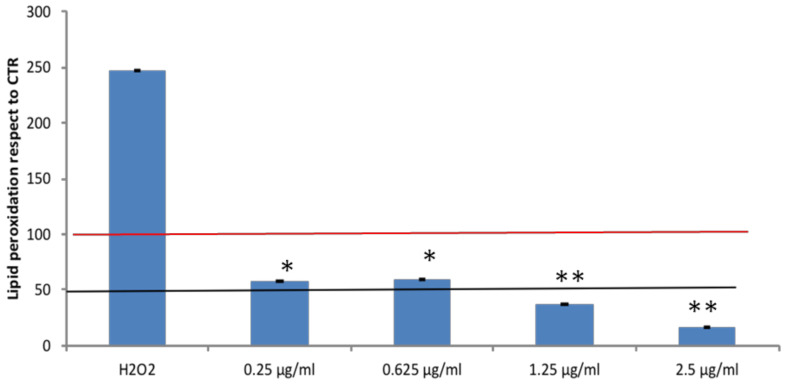
Effect of açai extract on lipid peroxidation in steatotic HepG2 cells pretreated with FAs. Lipid peroxidation levels were determined after 30 min incubation with H_2_O_2_ (50 µM) and then incubated with açai extract at different concentrations (0.25–0.625–1.25–2.5 µg/mL) for 24 h. Data are means ± SD of independent experiments (n = 3). * indicates *p* < 0.05; ** indicates *p* < 0.01 with respect to H_2_O_2_ pretreated cells. The red line is relative to cells pretreated to the sole FAs; the black line indicates the 50% of peroxidated lipid within cells.

Lipid peroxidation assay was performed on steatotic cells to investigate the protective effect of açai extract after oxidative stress induction with 50 µM H_2_O_2_. As shown in Figure 3, we noticed an effect of lipid peroxidation in the presence of only FAs exposition and successively a significant decrease was observed upon treatment of steatotic cells with açai extract at all the concentrations tested. As shown in Figure 2, the strongest effect was observed at 2.5 µg/mL, the same concentration able to reduce 50% in cell viability. For this reason, the treatment at 1.25 µg/mL seems to be the most effective (since a significant inhibition in lipid peroxidation levels was observed) and not related to cell death.

#### 3.2.3. Effects of Açai Extract on NF-kB Protein Expression in HepG2 Cells

**Figure 4:**
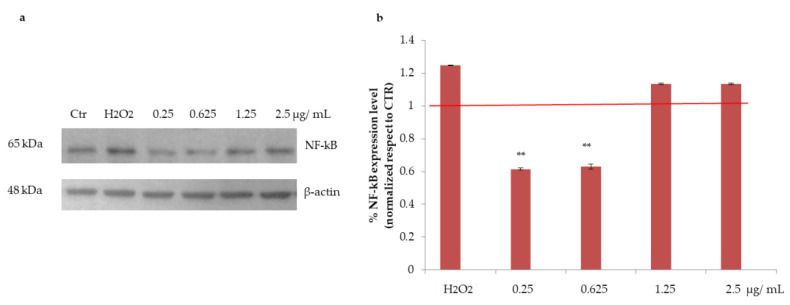
Effect of açai extract on nuclear NF-kB levels. (**a**) Western blot analysis of NF-kB levels and its relative densitometric analysis (**b**). The red line represents untreated cells. β-actin was used as an internal standard. Data shown are the means ± S.D. of independent experiments (n = 3). ** indicates *p* < 0.05.

Hydrogen peroxide (H_2_O_2_) challenge is known to induce oxidative stress as well as inflammation in HepG2 cells [15]. Thus, NF-kB, a well-established inflammatory marker, was analysed by Western blotting in HepG2 cells challenged with H_2_O_2_ and then treated with açai extract. As shown in Figure 4, a significant reduction in NF-kB level was observed upon treatment with açai extract (** *p* < 0.01 with respect to control) at the lowest concentrations tested (0.25 and 0.625 µg/mL). No protective effect was observed at the highest concentration tested, probably because of the cytotoxic effect of açai extract observed at these concentrations (Figure 2).

#### 3.2.4. Quali-Quantitative Analyses of Fat Levels in HepG2 and Adipocytes Cells Treated with Açai Extract

**Figure 5:**
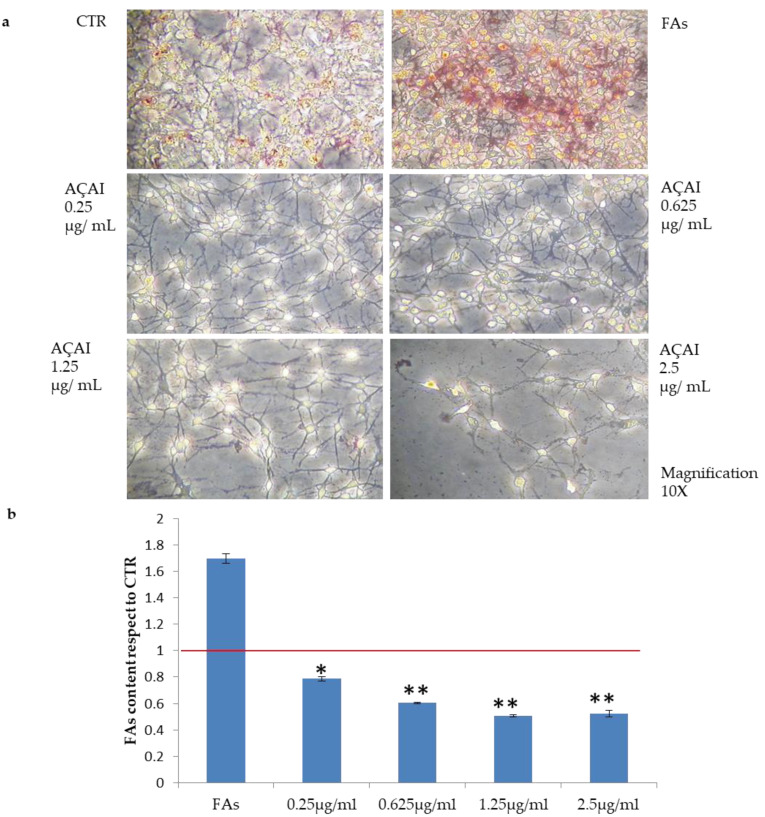
Effects of açai extract on in vitro induced steatosis on HepG2 cells. (**a**) Oil Red O staining images of steatotic HepG2 cells, and control (first image on the left). (**b**) Spectrophotometric quantification of lipids in HepG2 cells treated with açai extract at different concentrations (from 0.25 to 2.5 μg/mL) with respect to HepG2 control cells. Values are expressed as means ± S.D of independent experiments (n = 3). The red line is relative to control cells. * indicates *p* < 0.05; ** indicates *p* < 0.01.

Oil Red O staining showed that the treatment with oleic and linoleic acid mixture induced the accumulation of lipid droplets in almost all HepG2 cells, as shown in (Figure 4). As reported in Figure 5, açai extract was found to reduce lipid droplets formation even at the lowest concentration tested (0.25 µg/mL, * *p* < 0.05), reaching the highest inhibition activity at 0.625 µg/mL (** *p* < 0.01 with respect to control cells, reported as red line).

#### 3.2.5. Effects of Açai Extract on PPARs Protein Expression in HepG2 Cells

Adipogenesis requires the activation of several transcriptional factors, among which the peroxisome proliferator-activated receptor γ (PPARγ). As PPARα/γ [21] are at the center of the adipogenic cascade [22] and key terminal adipogenic regulators [23], their expression was evaluated in our experimental system. As shown in Figure 5, upon treatment with increasing amount of açai extract, PPARα/γ expression increased at the lowest concentration tested. On the other hand, the higher was the concentration of the extract, the lower the protein expression levels (Figure 5). This result may suggest that the impairment of cell viability induced by the extract in certain conditions affects also cell lipid metabolism (Figure 1). This evidence suggests that açai extract displays a hormetic action, similarly to other natural antioxidants (e.g., resveratrol) [24].

#### 3.2.6. Quali-Quantitative Analyses of Fat Levels in Adipocytes Cells Treated with Açai Extract

**Figure 6:**
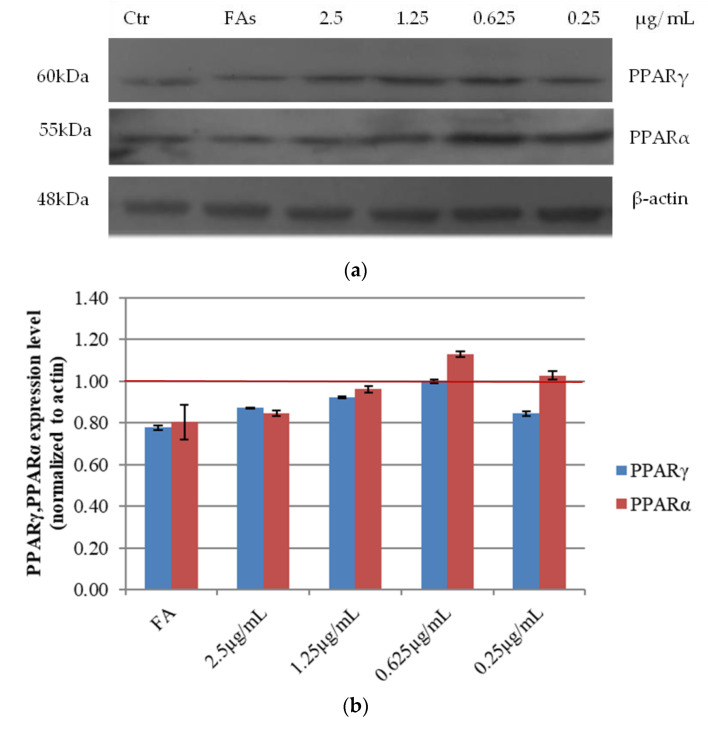
Western blot analysis of PPARα and PPARγ on HepG2 cells. (**a**) Western blot of steatotic HepG2 cells after incubation with increasing concentrations of açai extract. (**b**) Relative densitometric analysis of protein expression levels. Red line is referred to untreated cells. β-actin was used as an internal standard. Data are expressed as mean values ± SD of independent experiments (n = 3).

**Figure 7:**
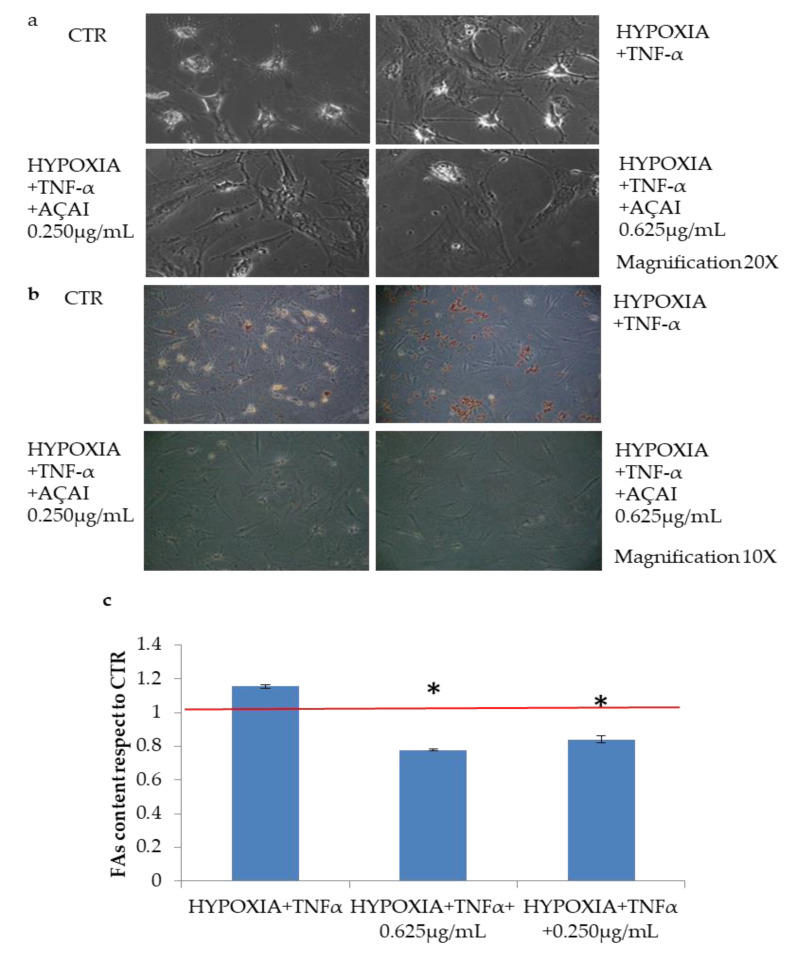
Effects of açai extract on in vitro induced persistent fat accumulation on adipocyte cells. (**a**) Images of adipocyte cells, either untreated and grown in physiological condition (CTR) or grown in hypoxic condition and treated with TNFα before staining, 20× magnification. (**b**) Oil Red O staining image of fat accumulation in adipocyte cells, 10× magnification. In physiological condition (CTR) and in the same hypoxic and treatment condition described for point (**a**). (**c**) Spectrophotometric quantification of lipid amount percentage of adipocyte cells treated with açai extract (0.25 and 0.625 μg/mL) with respect to CTR. Values are expressed as means ± S.D. of independent experiments (n = 3). The red line is relative to CTR that is grown in physiological condition. * indicates *p* < 0.05.

Lipid accumulation was detected with Oil Red staining on adipocyte cells treated with TNF-α (10 ng/mL) in hypoxic condition with respect to control cells (Figure 6). Interestingly, also on this cell model, açai extract efficiently reduced fat deposit (* *p* < 0.05), even at low concentrations reduced efficiently fat deposit (* *p* < 0.05) as shown in Figure 7.

## 4. Discussion

Resveratrol has been studied for 15 years and a wide number of products based on this active molecule are now available on the market. This paved the way to study other biomolecules or extracts containing polyphenols not only for the scientific interest itself, but also due to the potentialities of these compounds in aging, inflammatory diseases, and even cancer treatment [25]. The beneficial effect of the molecules investigated in the present study is strictly related to efficient extraction procedures. In the framework of this research two diverse solvents were used and compared, permitting to define the condition needed for efficient extraction of bioactives and their stability in long term storage. The GRAS solvents used were compared for their extraction capacity from desiccated açai and found to be interchangeable and to allow prolonged stability of açai extract. Thus, the antioxidant and hypolipidemic properties of açai extract on different in vitro cell models were evaluated. Our results showed that açai extract, especially at low doses, modulates lipid peroxidation in HepG2 cells and the response to oxidative stress through the reduction of nuclear NF-kB protein expression in vitro. The intracellular lipid amount was drastically reduced in the presence of the extract on NAFLD in vitro model. Similar to resveratrol, this effect is due to a PPARα-mediated mechanism. In fact, it has been reported that resveratrol analogues, such as pterostilbene, piceatannol, and resveratrol trimethyl ether, proved effective as lipid/lipoprotein lowering agents in hypercholesterolemic hamsters [26]. Specifically, pterostilbene was more effective as PPARα agonist and hypolipidemic agent than resveratrol [27]. Many antioxidant molecules, such as plant phenolics (including simple flavonoids, coumarins, stilbenes, hydrolyzable and condensed tannins, lignans, and lignins) are known to show a peculiar hormesis effect on cells, namely at low doses they show protective effects on lipid metabolism, whereas, at higher doses they induce cell toxicity [28]. Few studies reported that resveratrol inhibits cell viability in a dose- and time-dependent manner [29]. For these reasons we tested açai extract at low doses on different cell models. However, on HepG2 cells a slight cytotoxic effect according to ISO10993-5 was found only at the higher concentration tested (i.e., 2.5 µg/mL) adipocytes. To further evaluate the potential hypolipidemic activity of acai extracts, we set up a new model of fat accumulation, based on primary adipocytes cells to reproduce a condition better resembling localized adiposity also frequently named cellulitis in vivo. The cells were treated with TNFα in hypoxic conditions, to mimic persistent fat accumulation, considered as pathological condition.

Our results are in agreement with those recently published by de Freitas Carvalho and collaborators [30], who found a significant decrease of fat accumulation induced by açai extract both on in vitro and in vivo models. Further in vivo studies are necessary to establish a dose/response correlation, however also the specific application, in functional food, of food supplement or even cosmeceutical formulation (topical use), is relevant to assess the optimal active concentration. For instance, beneficial in vitro effects are demonstrated for resveratrol at concentrations ranging from 1 to 50 μM. However, these values are not directly correlated to in vivo outcomes. In fact, they do not consider potential interaction with proteins, lipoproteins, or other molecules and, thus, the actual free resveratrol concentration. In addition, bioavailability studies showed that, even after a high dose, only a small amount of resveratrol in its free form is present in plasma and thus highly concentrated treatments are required to reach serum levels corresponding to those necessary for the in vitro biological activities. In vivo effects were observed by Baur and collaborators [31], in a mouse model using a dosage of about 22.4 ± 0.4 mg kg^−1^ day^−1^, this value corresponds, based on the so-called body surface area (BSA) a parameter that is necessary for a correct dose conversion from mice to human, to about 128 mg for a man of 70 kg [32].

However, protective effects have been observed also at a lower resveratrol dose, (5.9 mg kg^−1^ day-1 in mice) that is equivalent to 33 mg for human use [33]. The literature reports previously discussed may help in understanding that the range of concentration studied here for açai extracts in vitro, are valuable to obtain a dose-range for further in vivo testing in relation to what already known for resveratrol analogues. In fact, it has been recently highlighted that, due to the interference of a few biological factors, the correlation between cellular and animal studies can be very tricky [34].

In Levy and Hasson’s research work (2020), it is recommended to collect pharmacokinetic data for the new compound also considering the lipophilicity/hydrophilicity of the active principle [35].

However, for each of the plant active extract, the specific formulation to be used has to be assessed before implementing protocols for preclinical studies.

Notably, the results presented in this study based on in vitro analyses of multiple cellular models, support açai extracts sound performances as antioxidant compound, with persistent effect in diverse storage condition, in reducing lipid peroxidation, and in limiting fat droplets deposit within the HepG2 and human adipocytes. To unravel the specific action mechanism further studies are needed, however a molecular approach via NF-kB through the well know adipogenic regulators PPARα/γ was here proposed, and Western blot data are consistent with the hypothesis formulated. Taken together, all these outcomes may suggest açai extract as a novel potential product for the treatment of lipid deposit accumulation that maybe exploited in novel nutraceuticals and/or topical cosmeceutical formulations or functional foods.

## 5. Conclusions

The results on diverse in vitro models, demonstrated that açai extract is a natural source of active and stable principles able to counteract oxidative stress and to inhibit lipid accumulation. These are considered among principal determinants of metabolic based syndromes and diseases. This could open the way for new açai-based nutraceutical or functional foods, and even cosmeceutical topical formulation, to support the treatment of these disorders.

## Figures and Tables

**Table 1:** Analysis of the protective effect of açai extract on ROS levels in HaCaT cells stressed by sodium arsenite (SA). Results are expressed as percentage of ROS in treated cells with respect to untreated cells. ND, not determined. Data shown are means ± S.D. of three independent experiments. * *p* < 0.05 with respect to control cells prior SA treatment; ** *p* < 0.01 with respect to control cells prior SA treatment; ^a^ *p* < 0.05, ^b^ *p* < 0.01, ^c^ *p* < 0.005 with respect to control cells after SA treatment.

Time	Storage Conditions	Ethanol		Isopropanol		SA Treatment
		H_2_O	PBS	H_2_O	PBS	
Time 0		118 ± 29	116 ± 20	117 ± 16	119 ± 21	155 ± 5 *
72 h	−20 °C	135 ± 47	166 ± 28	111 ± 8 ^a^	121 ± 14 ^a^	147 ± 1 **
	+4 °C	93 ± 12 ^a^	108 ± 14	123 ± 17	82 ± 13 ^a^	
	r.t. light	106 ± 15	94 ± 12 ^a^	106 ± 38	89 ± 15 ^b^	
	r.t. dark	130 ± 45	98 ± 3 ^a^	102 ± 7 ^b^	87 ± 15 ^b^	
	37 °C	137 ± 2	103 ± 31	78 ± 18 ^a^	101 ± 33	
7 days	−20 °C	77 ± 73	84 ± 36 ^a^	136 ± 20	93 ± 23 ^a^	150 ± 8 *
	+4 °C	216 ± 99	216 ± 37 ^a^	123 ± 18	109 ± 3 ^b^	
	r.t. light	40 ± 44	169 ± 31	164 ± 8 ^a^	89 ± 39	
	r.t. dark	50 ± 57	142 ± 23	124 ± 4 ^a^	96 ± 13 ^a^	
	37 °C	78 ± 39	208 ± 53	140 ± 36	105 ± 23	
14 days	−20 °C	158 ± 18	136 ± 11	70 ± 7 ^a^	59 ± 13 ^a^	147 ± 16 *
	+4 °C	114 ± 30	116 ± 7 ^a^	71 ± 2 ^c^	77 ± 17 ^a^	
	r.t. light	126 ± 15	147 ± 10	60 ± 37 ^a^	63 ± 13 ^c^	
	r.t. dark	114 ± 24	109 ± 5 ^b^	67 ± 18 ^c^	73 ± 9 ^c^	
	37 °C	143 ± 1	94 ± 11 ^c^	125 ± 7	84 ± 19 ^a^	
21 days	−20 °C	144 ± 23	168 ± 9 ^c^	151 ± 5	156 ± 42	140 ± 5 *
	+4 °C	57 ± 22 ^a^	194 ± 33 ^a^	181 ± 15 ^c^	170 ± 7 ^c^	
	r.t. light	198 ± 31	162 ± 12 ^c^	161 ± 11 ^b^	146 ± 14	
	r.t. dark	114 ± 3 ^a^	147 ± 10	150 ± 4 ^a^	147 ± 5	
	37 °C	159 ± 5 ^a^	149 ± 30	165 ± 30	137 ± 18	
70 days	−20 °C	147 ± 14	141 ± 1 ^a^	135 ± 10	128 ± 16	149 ± 4 *
	+4 °C	148 ± 17	158 ± 4 ^a^	149 ± 10	130 ± 22	
	r.t. light	126 ± 4 ^b^	145 ± 5	121 ± 26	157 ± 67	
	r.t. dark	129 ± 6 ^a^	118 ± 12 ^a^	133 ± 42	138 ± 50	
	37 °C	ND	ND	ND	ND	

## Data Availability

Not applicable.
